# Nitrogen Fixation in Denitrified Marine Waters

**DOI:** 10.1371/journal.pone.0020539

**Published:** 2011-06-07

**Authors:** Camila Fernandez, Laura Farías, Osvaldo Ulloa

**Affiliations:** 1 Departamento de Oceanografía and Centro de Investigación Oceanográfica en el Pacífico Sur-Oriental (COPAS), Universidad de Concepción, Concepción, Chile; 2 UPMC Université Paris 06 and CNRS, UMR 7621, LOMIC, Observatoire Océanologique, Banyuls/mer, France; Argonne National Laboratory, United States of America

## Abstract

Nitrogen fixation is an essential process that biologically transforms atmospheric dinitrogen gas to ammonia, therefore compensating for nitrogen losses occurring via denitrification and anammox. Currently, inputs and losses of nitrogen to the ocean resulting from these processes are thought to be spatially separated: nitrogen fixation takes place primarily in open ocean environments (mainly through diazotrophic cyanobacteria), whereas nitrogen losses occur in oxygen-depleted intermediate waters and sediments (mostly via denitrifying and anammox bacteria). Here we report on rates of nitrogen fixation obtained during two oceanographic cruises in 2005 and 2007 in the eastern tropical South Pacific (ETSP), a region characterized by the presence of coastal upwelling and a major permanent oxygen minimum zone (OMZ). Our results show significant rates of nitrogen fixation in the water column; however, integrated rates from the surface down to 120 m varied by ∼30 fold between cruises (7.5±4.6 versus 190±82.3 µmol m^−2^ d^−1^). Moreover, rates were measured down to 400 m depth in 2007, indicating that the contribution to the integrated rates of the subsurface oxygen-deficient layer was ∼5 times higher (574±294 µmol m^−2^ d^−1^) than the oxic euphotic layer (48±68 µmol m^−2^ d^−1^). Concurrent molecular measurements detected the dinitrogenase reductase gene *nifH* in surface and subsurface waters. Phylogenetic analysis of the *nifH* sequences showed the presence of a diverse diazotrophic community at the time of the highest measured nitrogen fixation rates. Our results thus demonstrate the occurrence of nitrogen fixation in nutrient-rich coastal upwelling systems and, importantly, within the underlying OMZ. They also suggest that nitrogen fixation is a widespread process that can sporadically provide a supplementary source of fixed nitrogen in these regions.

## Introduction

Fixed nitrogen is continuously being added to and removed from the ocean through processes mediated by microbial communities. Over large temporal and spatial scales, potential changes to the marine N inventory depend on the variability of biological nitrogen fixation and denitrification (the stepwise reduction of nitrate to N_2_; [1], [2]) plus anammox (the anaerobic ammonium oxidation with nitrite to N_2_ [3], [4]). The current view is that these processes are spatially disconnected [5], [6]. Marine nitrogen fixation, with a global rate of ∼150 Tg N y^−1^ [1], is thought to occur predominantly at the surface (and subsurface) of tropical oceans by the activity of diazotrophic phototrophs (such as the colonial cyanobacterium *Trichodesmium* [7]) and unicellular cyanobacteria [8], [9]. It is also performed, to a lesser degree, by non-photosynthetic diazotrophic bacterioplankton [10], [11]. In contrast, nitrogen losses, with global rates of ∼200 Tg N y^−1^ [1], are primarily the result of the activities of denitrifying and anammox bacteria [12]. Nitrogen losses occur mainly in sediments [13], anoxic basins [3], [4] and oxygen minimum zones (OMZs) [14], [15], [16]. The main oceanic OMZs are located in the eastern tropical Pacific Ocean and the Arabian Sea.

**Figure 1:**
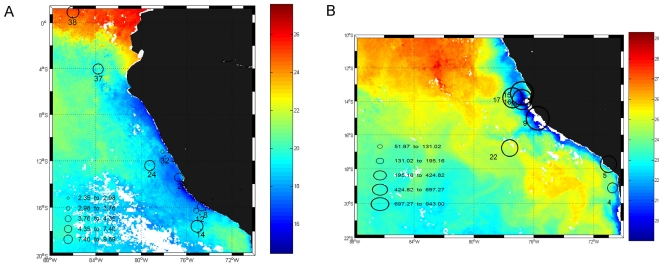
Location of the sampled stations and integrated N_2_ fixation rates. A) Knorr cruise (October–November 2005) and B) Galathea-3 expedition (February 2007). Stations are superimposed on daily N_2_ fixation rates (µmol m^−2^ d^−1^) integrated over the water column (black circles). Colour scale represents Sea Surface Temperature (SST °C).

Although it has been suggested that surface diazotrophic activity could be enhanced near areas with high rates of water-column denitrification [17] or even within oxygen-deficient waters [11], direct evidence for this process within OMZ regions has been missing. In this study, we report on direct N_2_ fixation rates and on the molecular diversity of the nitrogenase reductase gene *nifH* for waters of the eastern tropical South Pacific (ETSP) off Peru and northern Chile. This region of the ETSP is the site of permanent wind-driven coastal upwelling and contains a persistent, large and intense oxygen minimum zone (OMZ) at intermediate depths (80–400 m). It contributes a significant fraction of global marine nitrogen losses [15], [18], [19], [20]. Our results come from two oceanographic cruises, one carried out in October 2005 (R/V *Knorr*) and the other in February 2007 (Galathea-3, R/V *Vædderen*). Measurements were taken at stations located between 1.5°N and 20°S and at depths ranging from the surface to 120 m in 2005 and to 400 m in 2007 (Fig. 1).

## Results

**Figure 2:**
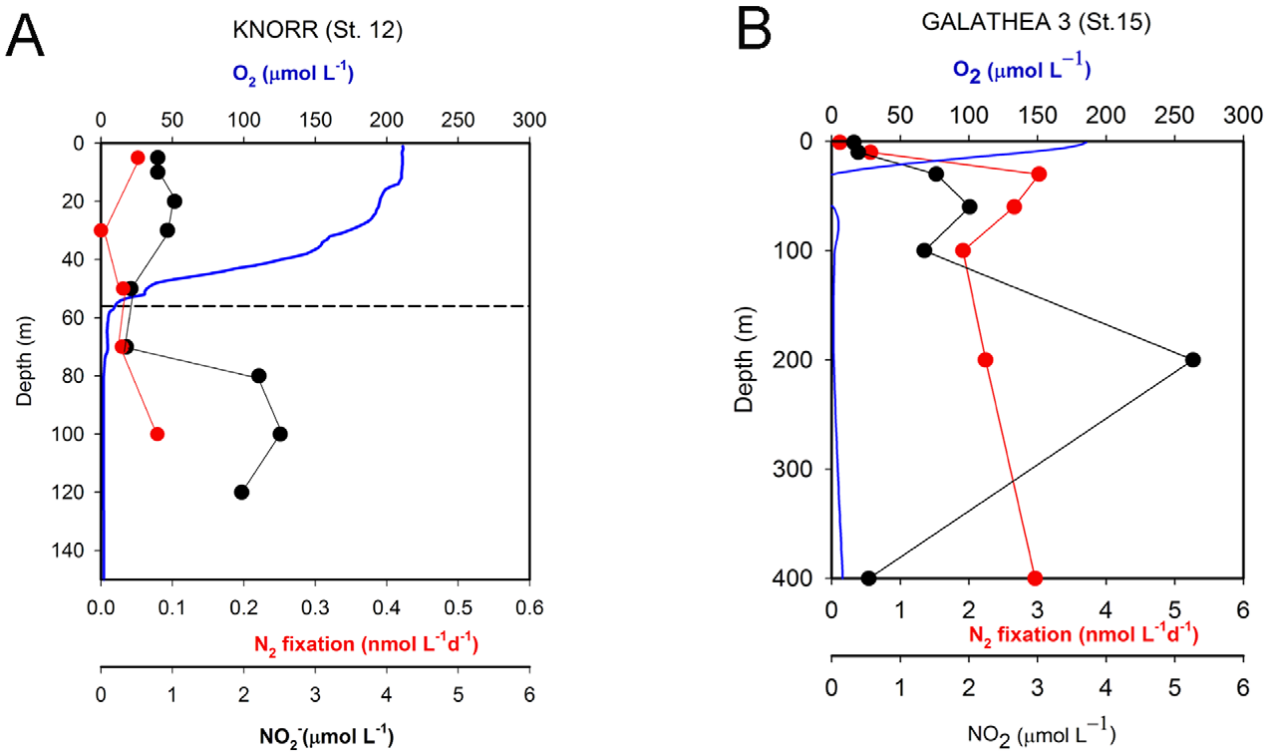
Selected N_2_ fixation profiles. Representative stations are plotted for cruises A) Knorr 2005 and B) Galathea-3 expedition 2007 carried out in Peruvian waters. Rates (nmol L^−1^ d^−1^) are represented in red dots. Full lines represent continuous oxygen profiles obtained from the CTD (upper cast). Nitrite concentrations (black points, µmol L^−1^) increase through the oxycline, forming the main secondary maximum at the core of the oxygen minimum zone. In this layer, N_2_O and NO_3_^−^ decrease, but a large amount of NO_2_^−^ accumulates.

Oceanographic conditions during both cruises showed active wind-driven coastal upwelling off northern Chile and Peru, as seen in the Sea Surface Temperature (SST) distribution (Fig. 1A, B). During Galathea-3 in 2007, SST values (17–24°C, Table 1) were higher than during the Knorr cruise in 2005 and bore the signature of the last phase of a moderately warm El Niño-Southern Oscillation (ENSO) event [21]. On both occasions, the signal of subsurface nutrient-rich oxygen-deficient waters (mainly associated with Equatorial Sub Surface Water (ESSW)), clearly appeared in the near surface waters as a result of the upwelling process. During both cruises, the vertical distribution of dissolved oxygen showed a sharp oxycline, with oxygen-deficient waters (in which nitrite starts accumulating to form a secondary nitrite maximum) reaching depths as shallow as 40 m (Fig. 2A and B). The oxygen-depleted zone extended from approximately 80 m to depths exceeding 400 m. During the 2007 cruise, essentially anoxic waters (<2 nmol L^−1^ O_2_) were measured at the core of the OMZ using the ultra-sensitive STOX oxygen microsensor [22]. Concentrations of dissolved inorganic N (DIN; NO_2_^−^, NO_3_^−^ and NH_4_^+^) during the 2005 and 2007 cruises were high. Average nitrate levels at the surface reached 7.8±5 (s.d.) and 5.5±3.8 µmol L^−1^ for the Knorr and Galathea-3 cruises, respectively, while average ammonium values reached 0.08±0.08 and 0.23±0.3 µmol L^−1^. The secondary nitrite maximum extended approximately from the upper boundary of the oxygen deficient zone to depths exceeding 120 m during the Knorr cruise and to depths of 400 m during the Galathea cruise (Fig. 2A and B). Molar N∶P nutrient ratios were generally below the canonical Redfield value of 16 in both surface waters (surface average was 6.3±4 versus 8.1±4.5 Table 1) and in deeper layers (average N∶P was 8.3±4.9 for the Knorr and 11.9±4.4 for the Galathea cruises, respectively). Exceptions were observed at stations 24 and 38 for the Knorr cruise and stations 9, 14 and 16 for the Galathea-3 expedition (Table 1). Likewise, values of P* (an index of the excess of inorganic phosphorous relative to inorganic nitrogen [17]) for the water column reflected conditions of nitrogen deficiency (0.99±0.7 and 0.59±0.6 in 2005 and 2007, respectively) indicating the vertical advection of denitrified water towards the surface.

**Table 1:** Geographical location and hydrographic features of the stations visited during cruises Knorr and Galathea 3.

Cruise	Station	Longitude (°E)	Latitude (°N)	SST^a^ (°C)	Surface NO_3_^−^ (µmol L^−1^)	SurfaceN∶P ratio	SurfaceP*^b^	Surface ρ^15^N_2_ (nmol N L^−1^ d^−1^)
**Knorr**	8/9	74.65	−15.91	15.0	9.6	5.7	1.69	0.06
**Knorr**	12	75.61	−16.28	15.9	10.3	7.4	0.67	0.03
**Knorr**	14	76.69	−17.68	17.2	1.5	1.5	1.39	0.04
**Knorr**	20	76.99	−13.3	15.3	7.4	3.8	1.4	0.27
**Knorr**	24	79.3	−12.25	17.1	18.4	12.4	0.25	0.11
**Knorr**	32	78.16	−10.99	16.3	12.9	7	1.02	0.03
**Knorr**	37/33	83.94	−3.59	18.5	8.9	11.13	0.72	0.01
**Knorr**	38	86.94	1.5	25.3	0.9	1.6	0.5	0.15
**Galathea3**	9	75.75	−15.5	21.1	10.9	11.55	−0.1	0.23
**Galathea3**	5	71.03	−18.5	23.9	4.1	4.2	0.23	0.05
**Galathea3**	4	70.76	−20.6	22.1	1.4	2.3	0.55	0.55
**Galathea3**	14	76.43	−14.39	17.7	10.7	13.05	0.1	n.d.^c^
**Galathea3**	22	77.62	−17.44	21.3	5	3.79	0.09	1.85
**Galathea3**	17	77.43	−14.16	20.6	4.9	6.26	−0.28	0.23
**Galathea3**	15	76.8	−13.87	19.8	9.29	9.66	0.22	0.12
**Galathea3**	16	76.79	−14.28	19.9	6.1	14.03	−0.33	0.2

a Sea Surface Temperature.

b P* represents an index of excess phosphorous compared to inorganic nitrogen [17].

c Not detected.

**Figure 3:**
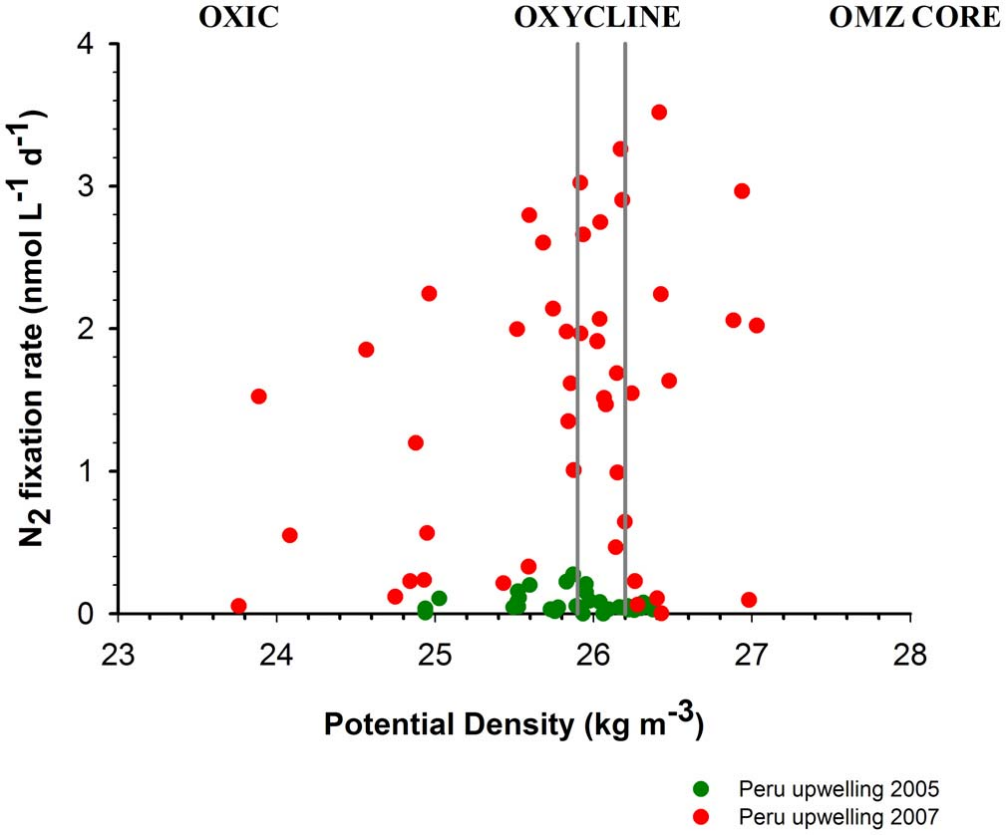
Nitrogen fixation rates of cruises Knorr 2007 and Galathea-3 2007. The nitrogen fixation data gathered for northern Chile and Peruvian upwelling in 2005 (Knorr cruise, green circles) and 2007 (Galathea 3 expedition, red circles) is plotted against potential density from surface oxic layer to the core of the Oxygen Minimum Zone.

N_2_ fixation occurred at a wide range of depths in both cruises and was detected in surface oxic and subsurface suboxic waters (Fig. 3). During the Knorr cruise (2005), N_2_ fixation rates in surface waters ranged between 0.01 and 0.27 nmol N L^−1^ d^−1^ (average 0.089±0.08 nmol N L^−1^ d^−1^, n = 17). In the upper oxycline, rates were in the same range as the surface values and maximum N fixing activities reached 0.2 nmol N L^−1^ d^−1^ (average 0.075±0.07 nmol N L^−1^ d^−1^ n = 8). Within the upper OMZ (sampling was only carried out to 120 m depth during this cruise), rates decreased to an average of 0.041±0.02 nmol N L^−1^ d^−1^. During the Galathea-3 expedition in 2007, rates of N_2_ fixation were over an order of magnitude higher than rates found in the previous cruise. Rates obtained in surface waters reached up to 2.34 nmol N L^−1^ d^−1^ (average 0.66±0.7 nmol N L^−1^ d^−1^; n = 10). An exceptionally high rate was detected at station 4 at 15 m depth (14 nmol N L^−1^ d^−1^) and was coincident with high NH_4_^+^ concentrations (0.7 µmol L^−1^). Rates in the upper oxycline reached maximum values of 3.26 nmol N L^−1^ d^−1^, with an average rate of 1.71±1.03 nmol N L^−1^ (n = 17). The deeper sampling in 2007 allowed us to detect active nitrogen fixation within the core of the OMZ and at a maximum depth of 400 m; these measurements considerably extended the currently accepted vertical and geographical distribution of marine nitrogen fixation. To our knowledge, this is the deepest water column measurement of N_2_ fixation to date. Rates obtained at the core of the OMZ were as high as 3.5 nmol N L^−1^ d^−1^, with an average value of 1.27±1.2 nmol N L^−1^ d^−1^ (n = 13). Overall, comparisons of both data sets show that the N_2_ fixation rates measured during the Galathea-3 cruise largely exceeded those measured in 2005, both in surface waters (0.07±0.07 versus 0.36±0.46 nmol N L^−1^ d^−1^ (e.g., Fig. 2A and B) and for integrated values down to 120 m (from 7.5±4.6 to 190±82.3 µmol m^−2^ d^−1^).

**Figure 4:**
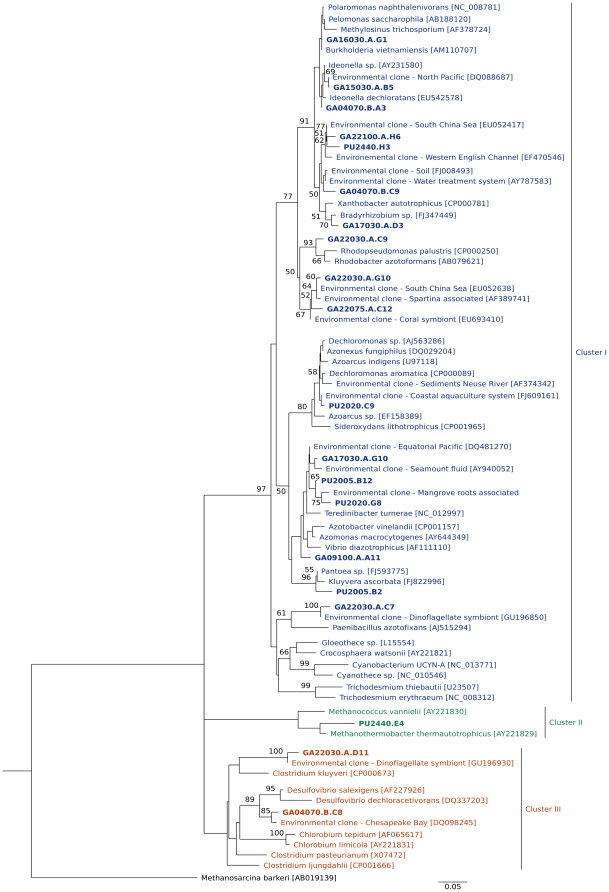
Maximum-likelihood phylogenetic tree of *nifH* predicted amino acid sequences obtained in the Peru coastal upwelling. Sequences for representative clones (≥95% identity at the nucleotide level) are given in bold (PU and GA indicate representative sequences for the Knorr and Galathea-3 cruises, respectively). Bootstrap support values (≥50%) for 1000 replications are shown at nodes. The scale bar indicates the number of sequence substitutions per site. The archaean *Methanosarcina barkeri* was used as an outgroup. Accession numbers for published sequences used to construct the phylogenetic tree are given in parenthesis. Additional information is given as Table S1, Table S2, Table S3, and Table S4.

To assess the community composition and distribution of diazotrophs, we amplified DNA sequences for the nitrogenase reductase gene *nifH*, which encodes for the metal protein of the nitrogenase enzyme complex. Positive amplifications were obtained for all the stations in which ^15^N_2_ fixation experiments were performed. Phylogenetic analyses of clone libraries obtained from selected stations in 2005 and 2007 (1125 valid *nifH* sequences) showed the existence of microorganisms with the genetic potential for N_2_ fixation (Fig. 4) at different depths (see Table S1, Table S2, Table S3, and Table S4). However, the diversity of the *nifH* genes for the 2005 cruise was very low compared to the diversity found in other marine systems [23], [24], and was especially low compared to what we found in 2007 (6 versus 14 phylotypes with 95% similarity at the nucleotide basis, respectively). Our *nifH* sequences fell within three of the four known clusters for this gene [25]. Most of our clones fell within Cluster I, which includes α, β and γ proteobacterial (as well as cyanobacterial) nitrogenases, and which has many marine representatives (Table S1, Table S2, Table S3, and Table S4). Importantly however, no sequences associated with cyanobacteria were found during our study, particularly within the euphotic zone. The rest of our sequences fell within Clusters II and III. These clusters include nitrogenases coming from the group Archaea, as well as diverse anaerobic microorganisms (such as the sulfate reducers chlorobiaceae and clostridia (Fig. 4)), but contain few marine representatives [26]. These molecular results thus suggest the presence of a diverse community of diazotrophs in the region during times of high N_2_ fixation rates. These results also indicate the need for further investigating the identity of the most important nitrogen fixers in these waters through, for example, *in situ* nitrogenase gene expression and stable-isotope probing studies, as well as culturing efforts.

## Discussion

Biological N_2_ fixation was detected during two cruises (2005 and 2007) off northern Chile and Peru using the ^15^N isotopic technique [27]. Rates obtained during both cruises were in the range of previously reported N_2_ fixation (Table S5), although values differed significantly (by an order of magnitude) between cruises. Phylogenetic diversity of the *nifH* sequences also varied greatly between cruises, in agreement with the biogeochemical rates of N_2_ fixation. Several aspects should be considered in the analysis of factors that govern this variability. First, hydrographic conditions differed between cruises: SST was higher during the Galathea-3 cruise than it was during the Knorr cruise, although we did not observe a clear correlation between the distribution of our N_2_ fixation rates and temperature. Second, significant differences were observed in P* and N∶P values (Student t-test values of p<0.05 in both cases, Table 1). However, the stoichiometry of the water column alone cannot account for the observed variability in our rates, as the data distributed over a vast range of P* values (see Fig. S1). Third, the vertical distribution of nitrogen fixation extended into the core of the OMZ, suggesting that this process might not respond solely to an excess of P compared to N in the surface waters of the ETSP [17] but that it could also be linked to varying levels of anoxia in the water column. Indeed our highest rates were obtained during the Galathea-3 expedition, when complete oxygen depletion was observed in the core of the OMZ. Unfortunately, we do not have STOX measurements for the 2005 cruise to compare the levels of oxygen-deficiency between cruises. Overall, our results suggest that N_2_ fixation acts as a transient process in denitrified marine waters. This characteristic has already been observed for other diazotrophic communities, such as the well-known bloom-forming cyanobacterium *Trichodesmium* in the North Pacific Subtropical gyre [28].

The overall input and potential significance of the N fixation process can be evaluated by integrating N_2_ fixation rates throughout the upper and the OMZ layers (see methods) and comparing them with the nitrogen losses. Daily integrated diazotrophic inputs from the surface to the 10-µmol O_2_ L^−1^ level at the base of the oxycline reached 48±68 µmol N m^−2^ d^−1^ in 2007. N_2_ fixation within suboxic waters (e.g., taken from the deepest level of the 1- µmol L^−1^ isoline to the deepest level of the average profile — 400 m for Galathea-3 cruise) revealed a contribution of 574±294 µmol N m^−2^ d^−1^ of newly-fixed N coming from the OMZ. These integrated input rates correspond to up to 5% of the N losses, which are estimated to be on average ∼11 mmol m^−2^ d^−1^ in the Peruvian OMZ [20]. However, the high variability observed between cruises, as well as reports of a methodological underestimation of nitrogen fixation rates with the conventional application of the ^15^N technique [29], suggest that higher and deeper N_2_ fixation fluxes, than those reported here are possible in the ETSP.

Some important aspects must be kept in mind when evaluating the potential of N_2_ fixation for offsetting N losses. First, while denitrification and anammox should be spatially confined to subsurface oxygen-deficient waters, N_2_ fixation is not. Our results are consistent with the idea that nitrogen fixation is enhanced in the surface layer via vertically transported N-deficient waters [17], but they also show that this process is not confined to areas adjacent to upwelling centers. Instead, nitrogen fixation actively occurs in coastal upwelling waters and can extend to depths within the core of the OMZ, but with significant time variability. On the other hand, the occurrence of active sporadic N_2_ fixation could locally increase the N∶P ratios of organic matter and could therefore affect the signature origin of DIN removed via denitrification. This possibility was suggested by the excess N_2_ data obtained during the 2005 Knorr cruise in the ETSP [30]. Complementary evidence also exists in the form of isotopic anomalies that differ from expectations for denitrification in OMZs [31], [32], [33]. For these cases, the re-mineralization of organic matter bearing the signature of nitrogen fixation has been suggested.

The marked difference between cruises in the diversity of *nifH* genes could indicate that high nitrogen fixation rates might not be the result of a single blooming diazotroph, but of a broader community. The distribution of our rates, which cover surface as well as subsurface and deep water with varying oxygen levels, also supports this idea. On the other hand, the fact that we found no *nifH* sequences associated with cyanobacteria in surface waters is consistent with previous surveys in the south-eastern border of the South Pacific gyre [34]. In those studies, extremely low abundances of group UCYN-A cyanobacteria were observed along with a total absence of large and group UCYN-B cyanobacterial diazotrophs. Also in agreement with our data, very low abundances of cyanobacterial *nifH* sequences were recently found at the redoxcline of the meromictic Lake Cadagnio, where other diazotrophs thrive [35]. In addition, a few of our sequences were grouped with microorganisms suspected to be reagent contaminants (e.g., β-proteobacteria [36], [37]. Thus, studies that unambiguously link function with taxonomic identity are needed.

Finally, although the temporal and spatial resolution of our study is not fully representative of the range of variability of N_2_ fixation within OMZs and coastal surface waters (e.g., the Knorr cruise was carried out in late spring, while the Galathea expeditions covered the late summer season), our measurements reveal a dynamic process with high temporal variability. Our molecular data also suggest that a diverse diazotrophic community can develop at certain times in the Peruvian upwelling ecosystem, for which oxygen-deficient conditions as well as persistent N removal [15], [20] may alleviate the inhibition of the nitrogenase enzymatic machinery by oxygen.

In summary, observations in the eastern tropical South Pacific demonstrate that significant diazotrophic activity occurs in oxic and subsurface denitrified waters, albeit with significant temporal variability. N_2_ fixation co-occurs with water column denitrification, a feature that has been described for sediments [38], [39]. Factors such as the level of oxygen-deficiency in the water column, hydrographic physical conditions (e.g., temperature) or nutrient ratios may be responsible for the observed variability between cruises. Further studies are needed to determine the transient nature of the phenomenon, the identity of the main diazotrophs, and whether nitrogen fixation occurs in other OMZ regions.

## Materials and Methods

Data presented here were obtained during two different cruises, the KN182-9 cruise (R/V *Knorr*; October-November 2005) and the Galathea-3 expedition (R/V *Vædderen*; March 2007). Both cruises covered the ETSP and particularly the Peruvian upwelling and northern Chile area (1.5°N to 20°S). Core parameters (including nutrients and dissolved oxygen), as well as biogeochemical variables (natural C and N isotopic composition, POC/ PON, Chl-a, DNA), were determined at all stations during these cruises.

All water samples were retrieved with 11-L Niskin bottles attached to a Conductivity-Temperature-Depth / rosette system (Seabird). For nutrient measurements, water was sampled with a 60 mL plastic syringe and filtered through a glass fiber filter (pore size 0.7 µm) into high-density polypropylene scintillation vials that were immediately frozen at −20°C. Samples were stored until laboratory analysis using an Alpkem® autoanalyzer (Knorr 2005 Cruise) or a manual colorimetric technique (Galathea-3 cruise) according to standard protocols for ammonium [40] and nitrate, nitrite and phosphate determination [41].

### ^15^N_2_ Fixation Experiments

Measurements of N_2_ fixation were performed in both cruises following the existing protocol for ^15^N_2_ trace addition experiments [27]. Incubations for N_2_ fixation were performed using 2-L tedlar gas-tight bags. These were equipped with inox (2005 cruise) or Teflon (2007 cruise) caps that included a silicone septum though which trace additions of ^15^N_2_ (99 atom%; CAMPRO SCIENTIFIC) were made with a gas-tight syringe at 2 mL gas L^−1^ of seawater. In all cases, samples were directly retrieved from the Niskin bottles using gas-tight Tygon tubes, which prevented contact with atmospheric oxygen and also prevented air-bubbles from entering the sample bags. For incubations using tedlar bags, the volumes and weights of filled bags were recorded at the beginning and at the end of the incubation process and real volumes were used in rate calculations. Possible permeability of tedlar to oxygen was reduced with double-layered tedlar. As bags were incubated under water, permeability (if it existed) should have been significantly reduced [42] and should not have exceeded 2 µmoles per liter of seawater per day. Since nitrogenase is oxygen sensitive [43], the effects of oxygen contamination should result in an underestimation (rather than an overestimation) of the true rate. Moreover, because all measurements were performed as ^15^N_2_ gas bubble injections, incomplete equilibration of isotopic gas during a standard incubation period might result in underestimations of N_2_ fixation rates [29]. Therefore, the actual rates of N_2_ fixation in this study could be higher than those reported.

In both cruises, incubations were performed on deck and lasted 24 h. Six deck incubators were maintained at sea surface temperature with light intensities ranging between 65 and 4% of incident light (Lee Filters®). Samples coming from below the 4% light level (Knorr and Galathea-3 cruises) were incubated in the dark in a thermo-regulated bath (Johnson Control®) or temperature-controlled incubator (Velp®) at *in situ* temperatures.

All incubations were terminated by gentle filtration onto 0.7 µm glass fiber filters (GF/F precombusted at 450°C; 12 h) using a vacuum (<100 mm Hg) or a peristaltic pump. Filters were dried at 60°C for 24 h and stored at 40°C until laboratory analysis by mass spectrometry. Once in the laboratory, filters were wrapped in tin cups and packed into pellets before analysis by continuous-flow isotope ratio mass spectrometry (IRMS delta plus, Thermo Finnigan®). Values given are a range of daily rates for all stations. Vertical profiles were separated in three distinct layers according to the potential density structure reported for the area [44] and corresponding to Subtropical Surface Water, Eastern South Pacific Intermediate Water and Equatorial Subsurface Water. Vertical integration was carried out by obtaining an average depth profile for each hydrographic layer (upper, oxycline and OMZ) and integrating within the specific depth range. Integrated values over each layer were then added to obtain an overall contribution of N through diazotrophy per cruise. The linear accumulation of ^15^N in particulate organic matter was tested using samples (5 to 80 m depth) obtained in central Chile in 2008 (Fig. S2). Results obtained during a time-series experiment showed increasing %^15^N in all samples, while particulate nitrogen (PN) remained relatively constant.

#### Community composition of diazotrophs

During the cruises Knorr (2005) and Galathea-3 (2007), water samples were collected at all stations for molecular characterization of the diazotroph community. Samples (up to 10 L) were successively filtered onto 3.0-µm (Isopore) and 0.22-µm (Sterivex-GV) pore-size filters, which were immediately covered with 2 mL of EDTA buffer and stored in liquid nitrogen until DNA extraction in the laboratory. Samples of both size fractions (> and <3 µm) were independently amplified by nested-Polymerase Chain Reaction (nested-PCR) with *nifH* primers (see Supplementary Information). The resulting PCR products from selected stations were cloned and sequenced to characterize the *nifH* sequence diversity.

#### Isolation and purification of nucleic acids

Lysozyme (50 mg mL^−1^) was added to the Sterivex filter and the filter unit incubated at 37°C for 45 min. Then, proteinase K (10 mg mL^−1^) and sodium dodecyl sulfate (SDS, 20%) were added, and the filter unit incubated at 50°C for 2 h. In the case of the Isopore filter, the procedure was similar but carried out in a 15-mL Falcon tube with 10% SDS. The lysates were then extracted once with phenol-chloroform-isoamyl alcohol (25∶24∶1; pH 8) and once with chloroform-isoamyl alcohol (24∶1). The samples were precipitated with isopropanol and sodium acetate (3 mol L^−1^, pH 5.2), and the pellets were washed with ethanol and then re-suspended with HPLC water. Nucleic acid extracts were stored at −20°C.

#### *nifH* PCR

To amplify *nifH* genes, a nested Polymerase Chain Reaction (nested-PCR) was performed. First, 1 µL of a 20 ng µL^−1^ of DNA was added to a PCR containing 1× PCR buffer (GoTaq, Promega), 2 mmol L^−1^ MgCl_2_, 0.2 mmol L^−1^ deoxynucleoside triphosphates, 1 µmol L^−1^ of *nifH4* (5′-TTY TAY GGN AAR GGN GG-3′ ), 1 µmol L^−1^ *nifH3* (5′-ATR TTR TTN GCN GCR TA-3′) [45] primers and 0.5 U of Taq DNA polymerase (GoTaq, Promega). All of the reagents were prepared with Dnase- and Rnase-free water. Thermal cycling for the first PCR was performed as follows: 5 min at 94°C, 30 cycles of 1 min at 94°C, 1 min at 55°C, and 1 min at 72°C, followed by a final extension step of 10 min at 72°C. After that, the samples were purified with the E.Z.N.A. Cycle Pure Kit (Omega Bio-Tek). Under the same conditions, but using a different pair of degenerate primers (*nifH1* and *nifH2* [46]), an aliquot of 1 µL of this purified PCR product was added to a nested PCR. For the nested PCR, the only difference was the annealing temperature, which in this case was 57°C. Subsequently, 50 µL of each PCR reaction were purified with the E.Z.N.A. Cycle-Pure Kit (Omega Bio-Tek). In both PCRs, a total of six negative controls were run to preclude false positives. The purified PCR products were cloned using the pGEM-T Easy vector systems according to the manufacturer's instructions (Promega). Sequencing was done by Macrogen Inc. (Korea).

#### Phylogenetic analysis

A total of 1125 valid sequences were obtained in this study, 437 for the Knorr cruise and 688 for the Galathea expedition (more detailed information is given in Table S1, Table S2, Table S3, and Table S4). Representative clones, one from each of the different phylogenetic groups identified based on ≥95% nucleotide similarity (Table S1, Table S2, Table S3, and Table S4), were used to construct the phylogenetic trees. Sequence alignment in the amino acid space was performed with MUSCLE 3.6 [47]. The maximum-likelihood phylogenetic tree in Fig. 4 was constructed with PhyML [48] using the default parameters in the program *Bosque* [49]. Percentages of Bootstrap support values (≥50) based on 1000 replications are shown at the nodes. Sequence data were deposited in the GenBank database under accession numbers HM801148 to HM801841.

## Supporting Information

Figure S1 N_2_ fixation rates versus P* (and index of the excess inorganic phosphorous relative to inorganic nitrogen [17]). Rates of N_2_ fixation were distributed across a wide range of P* values during the Knorr and Galathea-3 cruises.
(TIF)

Figure S2 Time course experiments of nitrogen fixation rates carried out in the upwelling system off central Chile (36°S) in 2008. Data shows (A) an accumulation of %^15^N in all samples over time and (B) A relatively constant trend in particulate nitrogen (PN) during the same experiments. Samples were obtained at 5 m (Times Series 1), 80 m (Time Series 2), 15 m depth (Time Series 3) and 30 m depth (Time Series 4).
(TIF)

Table S1 Operational Taxonomic Units (OTUs; with 95% similarity at the nucleotide basis [50]–[52]) and representative *nifH* sequences for the Knorr cruise (2005).
(DOC)

Table S2 Distribution of the different OTUs found at each station and depth during the Knorr cruise.
(DOC)

Table S3 Operational Taxonomic Units (OTUs; with 95% similarity at the nucleotide basis [52]–[58]) and representative sequences for the Galathea 3 cruise.
(DOC)

Table S4 Distribution of the different OTUs found at each station and depth during the Galathea-3 cruise.
(DOC)

Table S5 Comparison between water column N_2_ fixation rates obtained from the literature ([8], [10], [35], [59]–[64]) and values obtained in this study. The table shows previously published rates for unicellular diazotrophs as well as rates obtained during this study. Colonial diazotrophic cyanobacteria were not included in the table because they were not detected in the study area. The listed techniques are: ARA (Acetylene Reduction Assay), NA (Nitrogenase Activity) and ^15^N_2_ (Stable isotope tracer technique). Rates reported as hourly estimates (*) were transformed into daily rates regardless of potential daily periodicity in unicellular diazotrophs.
(DOC)

## References

[1] Codispoti LA (2007). An oceanic fixed nitrogen sink exceeding 400 Tg N a^−1^ vs. the concept of homeostasis in the fixed-nitrogen inventory.. Biogeosciences.

[2] Falkowski PG (1997). Evolution of the nitrogen cycle and its influence on the biological sequestration of CO_2_ in the ocean.. Nature.

[3] Dalsgaard T, Canfield DE, Petersen J, Thamdrup B, Acuña-González J (2003). N_2_ production by the anammox reaction in the anoxic water column of Golfo Dulce, Costa Rica.. Nature.

[4] Kuypers MM, Sliekers AO, Lavik G, Schmid M, Jorgensen BB (2003). Anaerobic ammonium oxidation by anammox bacteria in the Black Sea.. Nature.

[5] Berman-Frank I, Chen Y, Gao Y, Fennel K, Follows MJ, Capone D, Bronk D, Mulholland M, Carpenter EJ (2008). Feedback between the nitrogen, carbon and oxygen cycles.. Nitrogen in the marine environment.

[6] Gruber N, Follows F, Oguz T (2004). The dynamics of the marine nitrogen cycle and its influence on atmospheric CO_2_ variations.. The ocean carbon cycle and climate. NATO ASI Series.

[7] Capone DG, Burns JA, Montoya JP, Subramaniam A, Mahaffey C (2005). Nitrogen fixation by *Trichodesmium* spp.: An important source of new nitrogen to the tropical and subtropical North Atlantic Ocean.. Global Biogeochem Cy.

[8] Montoya JP, Holl CM, Zehr JP, Hansen A, Villareal TA (2004). High rates of N_2_ fixation by unicellular diazotrophs in the oligotrophic Pacific Ocean.. Nature.

[9] Zehr JP, Waterbury JB, Turner PJ, Montoya JP, Omoregie E (2001). Unicellular cyanobacteria fix N_2_ in the subtropical north Pacific Ocean.. Nature.

[10] Falcón LI, Carpenter EJ, Cipriano F, Bergman B, Capone DG (2004). N_2_ fixation by unicellular bacterioplankton from the Atlantic and Pacific Oceans: phylogeny and in situ rates.. Appl Environ Microb.

[11] Riemann L, Farnelid H, Steward GF (2010). Nitrogenase genes in non-cyanobacterial plankton: prevalence, diversity and regulation in marine waters.. Aquat Microb Ecol.

[12] Jetten MSM, Wagner M, Fuerst J, van Loosdrecht M, Kuenen G (2001). Microbiology and application of the anaerobic ammonium oxidation (anammox) process.. Curr Opin Biotech.

[13] Thamdrup B, Dalsgaard T (2002). Production of N_2_ through anaerobic ammonium oxidation coupled to nitrate reduction in marine sediments.. Appl Environ Microb.

[14] Kuypers MMM, Lavik G, Woebken D, Schmid M, Fuchs BM (2005). Massive nitrogen loss from the Benguela upwelling system through anaerobic ammonium oxidation.. PNAS.

[15] Thamdrup B, Dalsgaard T, Jensen MM, Ulloa O, Farías L (2006). Anaerobic ammonium oxidation in the oxygen-deficient waters off northern Chile.. Limnol Oceanogr.

[16] Ward BB, Devol AH, Rich JJ, Chang BX, Bulow SE (2009). Denitrification as the dominant nitrogen loss process in the Arabian Sea.. Nature.

[17] Deutsch C, Sarmiento JL, Sigman DM, Gruber N, Dunne JP (2007). Spatial coupling of nitrogen inputs and losses in the ocean.. Nature.

[18] Codispoti LA, Packard TT (1980). Denitrification rates in the eastern South Pacific.. J Mar Res.

[19] Copin-Montégut C, Raimbault P (1994). The Peruvian upwelling near 15°S in August 1986. Results of continuous measurements of physical and chemical properties between 0 and 200 m depth.. Deep Sea Res I.

[20] Hamersley MR, Lavik G, Woebken D, Rattray JE, Lam P (2007). Anaerobic ammonium oxidation in the Peruvian oxygen minimum zone.. Limnol Oceanogr.

[21] McPhaden MJ (2008). Evolution of the 2006–2007 El Niño: the role of intraseasonal to interannual time scale dynamics.. Adv Geosci.

[22] Revsbech NP, Larsen LH, Gundersen J, Dalsgaard T, Ulloa O (2009). Determination of ultra-low oxygen concentrations in oxygen minimum zones by the STOX sensor.. Limnol Oceanogr-Meth.

[23] Mehta MP, Butterfield DA, Baross JA (2003). Phylogenetic diversity of nitrogenase (*nifH*) genes n deep sea and hydrothermal vent environments of the Juan Fuca Ridge.. Appl Environ Microb.

[24] Zehr JP, Jenkins BD, Short SM, Steward GF (2003). Nitrogenase gene diversity and microbial community structure: a cross-system comparison.. Environ Microbiol.

[25] Chien YT, Zinder SH (1996). Cloning, functional organization, transcript studies, and phylogenetic analysis of the complete nitrogenase structural genes (nifHDK2) and associated genes in the archaeon *Methanosarcina barkeri* 227.. J Bacteriol.

[26] Zehr J, Church MJ, Moisander PH, Neretin LN (2006). Diversity, distribution and biogeochemical significance of nitrogen-fixing microorganisms in anoxic and suboxic ocean environments.. Past and present water column anoxia.

[27] Montoya J, Voss M, Kähler P, Capone D (1996). A simple, high-sensitivity tracer assay for N_2_ Fixation.. Appl Environ Microb.

[28] Dore JE, Letelier RM, Church MJ, Lukas R, Karl DM (2008). Summer phytoplankton blooms in the oligotrophic North Pacific Subtropical Gyre: Historical perspective and recent observations.. Prog Oceanogr.

[29] Mohr W, Groβkopf T, Wallace DWR, LaRoche J (2010). Methodological Underestimation of Oceanic Nitrogen Fixation Rates.. PLoS ONE.

[30] Chang BX, Devol AH, Emerson SR (2010). Denitrification and the nitrogen gas excess in the eastern tropical South Pacific oxygen deficient zone.. Deep Sea Res I.

[31] Brandes JA, Devol AH, Yoshinari T, Jayakumar DA, Naqvi SWA (1998). Isotopic composition of nitrate in the central Arabian Sea and eastern tropical North Pacific: A tracer for mixing and nitrogen cycles.. Limnol Oceanogr.

[32] DePol-Holz R, Robinson RS, Hebben D, Sigman D, Ulloa O (2009). Controls on sedimentary nitrogen isotopes along the Chilean margin.. Deep Sea Res II.

[33] Sigman DM, Granger J, DiFiore P, Lehmann M, Ho R (2005). Coupled nitrogen and oxygen isotope measurements of nitrate along the eastern North Pacific margin.. Global Biogeochem Cy.

[34] Bonnet S, Guieu C, Bruyant F, Prasil O, van Wambeke F (2008). Nutrient limitation of primary productivity in the Southeast Pacific (BIOSOPE cruise).. Biogeosciences.

[35] Halm H, Musat N, Lam P, Langlois R, Musat F (2009). Co-occurrence of denitrification and nitrogen fixation in a meromictic lake, Lake Cadagno (Switzerland).. Environ microbiol.

[36] Goto M, Ando S, Hachisuka Y, Yoneyama T (2005). Contamination of diverse *nifH* and *nifH*-like DNA into commercial PCR primers.. FEMS Microbiol Lett.

[37] Zehr JP, Crumbliss LL, Church MJ, Omoregie EO, Jenkins BD (2003). Nitrogenase genes in PCR and RT-PCR reagents: implications for studies of diversity of functional genes.. Biotechniques.

[38] Fulweiler RW, Nixon SW, Buckley BA, Granger SL (2007). Reversal of the net dinitrogen gas flux in coastal marine sediments.. Nature.

[39] Welsh D (2000). Nitrogen fixation in seagrass meadows: Regulation, plant-bacteria interactions and significance to primary productivity.. Ecol Lett.

[40] Holmes RH, Aminot A, Kérouel R, Hooker BA, Peterson BJ (1999). A simple and precise method for measuring ammonium in marine and freshwater ecosystems.. Can J Fish Aquat Sci.

[41] Parsons TR, Maita Y, Lalli CM (1984). A manual of chemical and biological methods for seawater analysis.

[42] Hansen JW, Thamdrup B, Jorgensen BB (2000). Anoxic incubation of sediment in gas-tight plastic bags: a method for biogeochemical process studies.. Mar Ecol-Progr Ser.

[43] Paerl HW, Zehr J, Kirchman DL (2000). Marine nitrogen fixation. Microbial Ecology of the Oceans.

[44] Farías L, Paulmier A, Gallegos M (2007). Nitrous oxide and N-nutrient cycling in the oxygen minimum zone off northern Chile.. Deep Sea Res I.

[45] Zani S, Mellon MT, Collier JL, Zehr JP (2000). Expression of *nifH* genes in natural microbial assemblages in Lake George, New York, detected by Reverse Transcriptase PCR.. Appl Environ Microb.

[46] Zehr J, McReynolds LA (1989). Use of degenerate Oligonucleotides for amplification of the *nifH* gene from the marine cyanobacterium *Trichodesmium thiebaitii*.. Appl Environ Microb.

[47] Edgar RC (2004). MUSCLE: multiple sequence alignment with high accuracy and high throughput.. Nucleic Acids Res.

[48] Guidon S, Gascuel O (2003). A simple, fast, and accurate algorithm to estimate large phylogenies by maximum likelihood.. Syst Biol.

[49] Ramírez-Flandes S, Ulloa O (2008). Bosque: integrated phylogenetic analysis software.. Bioinformatics.

[50] Steward G, Jenkins B, Ward B, Zehr J (2004). Development and testing of a DNA macroarray to assess nitrogenase (nifH) gene diversity.. Appl Environ Microb.

[51] Pinto-Tomas AA, Anderson MA, Suen G, Stevenson DM, Chu FST (2009). Symbiotic nitrogen fixation in the fungus gardens of leaf-cutter ants.. Science.

[52] Yang JC, Madupu R, Durkin AS, Ekborg NA, Pedamallu CS (2009). The complete genome of Teredinibacter turnerae T7901: an intracellular endosymbiont of marine wood-boring bivalves (shipworms).. PLoS ONE.

[53] Kopke M, Held C, Hujer S, Liesegang H, Wiezer A Clostridium Ijungdahlii represents a microbial production platform based on syngas.. PNAS.

[54] Choo Q, Samian M, Najimudin N (2003). Phylogeny and characterization of three nifH-homologous genes from Paenibacillus azotofixans.. Appl Environ Microb.

[55] Blaha D, Sanguin H, Robe P, Nalin R, Bally R (2005). Physical organization of phytobeneficial genes nifH and ipdC in the plant growth-promoting rhizobacterium Azospirillum lipoferum 4VI.. FEMS Microbiol Lett.

[56] Auman A, Speake C, Lidstrom M (2001). nifH sequences and nitrogen fixation in type I and type II methanotrophs.. Appl Environ Microb.

[57] Noar JD, Buckley DH (2009). Ideonella azotifigens sp. nov., an aerobic diazotroph of the Betaproteobacteria isolated from grass rhizosphere soil, and emended description of the genus Ideonella.. Int J Syst Evol Micr.

[58] Nzoue A, Miche L, Klonowska A, Laguerre G, deLajudie P (2009). Multilocus sequence analysis of bradyrhizobia isolated from Aeschynomene species in Senegal.. Syst Appl Microbiol.

[59] Needoba JA, Foster RA, Sakamoto C, Zehr JP, Johnson KS (2007). Nitrogen fixation by unicellular diazotrophic cyanobacteria in the temperate oligotrophic North Pacific Ocean.. Limnol Oceanogr.

[60] Montoya JP, Voss M, Capone CG (2007). Spatial variation in N2-fixation rate and diazotrophs activity in the Tropical Atlantic.. Biogeosciences.

[61] Dore JE, Brum JR, Tupas LM, Karl DM (2002). Seasonal and interannual variability in sources of nitrogen supporting export in the oligotrophic subtropical North Pacific Ocean.. Limnol Oceanogr.

[62] Zehr JP, Turner PJ (2001). Nitrogen fixation: Nitrogenase genes and gene expression.. Method Microbiol.

[63] Zehr JP, Montoya JP, Jenkins BD, Hewson I, Mondragon E (2007). Experiments linking nitrogenase gene expression to nitrogen fixation in the North Pacific subtropical gyre.. Limnol Oceanogr.

[64] Raimbault P, Garcia N (2008). Evidence for efficient regenerated production and dinitrogen fixation in nitrogen-deficient waters of the South Pacific Ocean: impact on new and export production estimates.. Biogeosciences.

