# Supplementary figures and images for: Nitrogen Fixation in Denitrified Marine Waters
Source: PLoS One. 2011 Jun 7;6(6):e20539. doi: 10.1371/journal.pone.0020539 (PMC3110191; doi:10.1371/journal.pone.0020539)

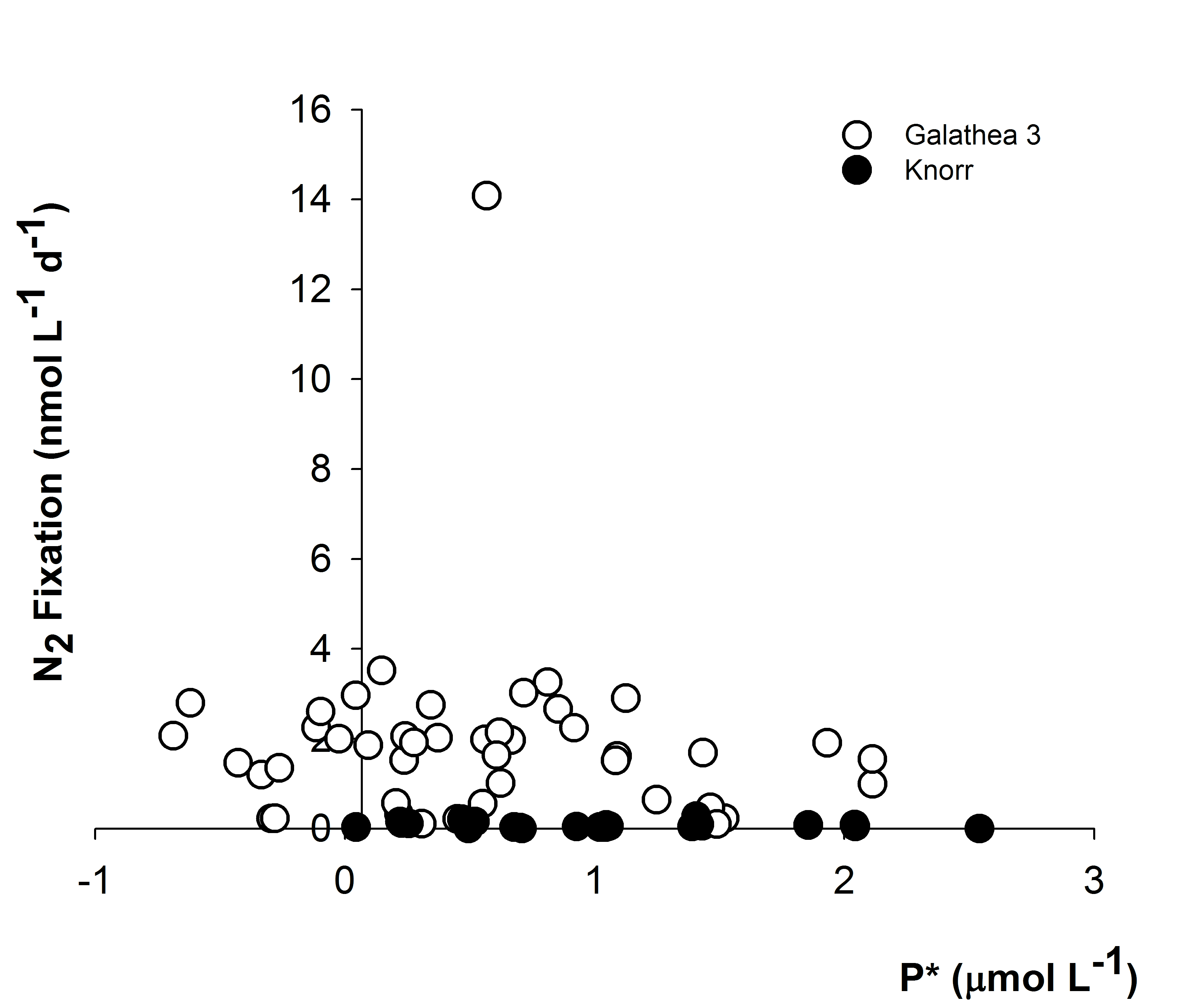

Supplement: Figure S1 — N2 fixation rates versus P* (and index of the excess inorganic phosphorous relative to inorganic nitrogen [17]). Rates of N2 fixation were distributed across a wide range of P* values during the Knorr and Galathea-3 cruises. (TIF) [file pone.0020539.s001.tif]

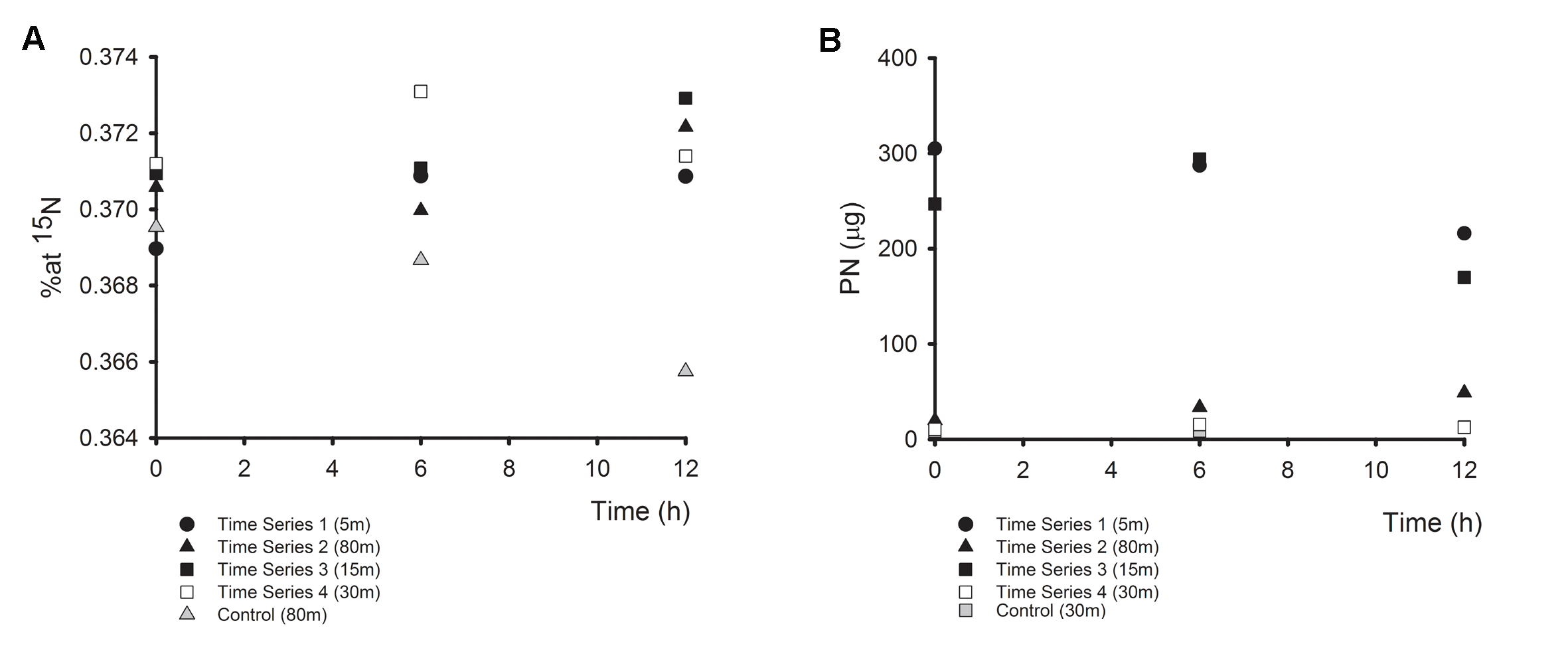

Supplement: Figure S2 — Time course experiments of nitrogen fixation rates carried out in the upwelling system off central Chile (36°S) in 2008. Data shows (A) an accumulation of %15N in all samples over time and (B) A relatively constant trend in particulate nitrogen (PN) during the same experiments. Samples were obtained at 5 m (Times Series 1), 80 m (Time Series 2), 15 m depth (Time Series 3) and 30 m depth (Time Series 4). (TIF) [file pone.0020539.s002.tif]
