# Supplementary material for: Nitrogen Fixation in Denitrified Marine Waters
Source: PLoS One. 2011 Jun 7;6(6):e20539. doi: 10.1371/journal.pone.0020539 (PMC3110191; doi:10.1371/journal.pone.0020539)
Supplement: Table S1 — Operational Taxonomic Units (OTUs; with 95% similarity at the nucleotide basis [50]–[52]) and representative nifH sequences for the Knorr cruise (2005). (DOC) [file pone.0020539.s003.doc]

| OTU | *nifH* Cluster | Representative sequence | N (total/unique seq.) | Closest Cultured | Accession Number | E Value | Taxa | Reference |
| --- | --- | --- | --- | --- | --- | --- | --- | --- |
| 1 | II | PU2440.E4 | 6/3 | *Methanothermobacter thermautotrophicus* AY221829.1 | AY221829 | 2.00e-143 | Archaea; Euryarchaeota; Methanobacteria; Methanobacteriales;Methanobacteriaceae; Methanothermobacter. | Steward et al. 2004 [50] |
| 2 | I | PU2440.H3 | 376/209 | ﻿*Xanthobacter autotrophicus* Py2 | ﻿CP000781 | 5.02e-119 | Bacteria; Proteobacteria; Alphaproteobacteria; Rhizobiales;Xanthobacteraceae; Xanthobacter | Gilbert et al. (unpublished) |
| 3 | I | PU2020.C9 | 5/5 | *Dechloromonas* sp. | AJ563286 | 1.96e-118 | Bacteria; Proteobacteria; Betaproteobacteria; Rhodocyclales; Dechloromonas | Minerdi et al. (unpublished) |
| 4 | I | PU2005.B2 | 45/23 | *Pantoea* sp. | FJ593775 | 1.61e-100 | Bacteria; Proteobacteria; Gammaproteobacteria; Enterobacteriales;Enterobacteriaceae; Pantoea | Pinto-Tomas et al. 2009 [51] |
| 5 | I | PU2005.B12 | 3/2 | *Azomonas macrocytogenes* | AY644349 | 3.11e-103 | Bacteria; Proteobacteria; Gammaproteobacteria; Pseudomonadales; Pseudomonadaceae; Azomonas | Seibold & Priefer (unpublished) |
| 6 | I | PU2020.G8 | 2/2 | *﻿Teredinibacter turnerae* | ﻿CP001614 | 3.11e-103 | Bacteria; Proteobacteria; Gammaproteobacteria; Alteromonadales; | Yang et al. 2009 [52] |
| Total |  |  | 437/244 |  |  |  |  |  |

Table S1: Operational taxonomic units (OTUs; with 95% similarity at the nucleotide basis[50-52]) and representative *nifH* sequences for the Knorr cruise (2005).
