# Supplementary material for: Nitrogen Fixation in Denitrified Marine Waters
Source: PLoS One. 2011 Jun 7;6(6):e20539. doi: 10.1371/journal.pone.0020539 (PMC3110191; doi:10.1371/journal.pone.0020539)
Supplement: Table S2 — Distribution of the different OTUs found at each station and depth during the Knorr cruise. (DOC) [file pone.0020539.s004.doc]

Table S2: Distribution of the different OTUs found at each station and depth during the Knorr cruise.

| Station | Depth (m) | OUT 1 | OUT 2 | OUT 3 | OUT 4 | OUT 5 | OUT 6 |
| --- | --- | --- | --- | --- | --- | --- | --- |
| 8 | 50 |  | x |  |  |  | x |
| 20 | 5 |  | x | x | x | x |  |
| 20 | 20 |  | x | x |  |  | X |
| 24 | 20 | x |  |  |  |  |  |
| 24 | 40 |  | x |  |  |  |  |
| 32 | 30 |  | x |  |  |  |  |
| 37 | 30 |  | x |  |  |  |  |
| 37 | 50 |  | x |  |  |  |  |
