# Supplementary material for: Nitrogen Fixation in Denitrified Marine Waters
Source: PLoS One. 2011 Jun 7;6(6):e20539. doi: 10.1371/journal.pone.0020539 (PMC3110191; doi:10.1371/journal.pone.0020539)
Supplement: Table S3 — Operational Taxonomic Units (OTUs; with 95% similarity at the nucleotide basis [52]–[58]) and representative sequences for the Galathea 3 cruise. (DOC) [file pone.0020539.s005.doc]

Table S3: Operational Taxonomic Units (OTUs; with 95% similarity at the nucleotide basis [52-58]) and representative sequences for the Galathea 3 cruise.

| OTU | *nifH* Cluster | Representative sequence | N (total/unique seq.) | Closest Cultured | Accession number | E value | Taxa | Reference |
| --- | --- | --- | --- | --- | --- | --- | --- | --- |
| 1 | III | GA22030.A.D11 | 8/5 | *Clostridium ljungdahlii* | CP001666 | ﻿5.54e-62 | Bacteria; Firmicutes; Clostridia; Clostridiales; Clostridiaceae; Clostridium. | Kopke et al. (2010) [53] |
| 2 | III | GA04070.B.C8 | 8/5 | *Desulfovibrio salexigens* | CP001649 | ﻿6.4e-87 | Bacteria; Proteobacteria; Deltaproteobacteria; Desulfovibrionales; Desulfovibrionaceae; Desulfovibrio | Lucas et al. (unpublished) |
| 3 | I | GA22030.A.C7 | 10/8 | *Paenibacillus azotofixans* | AJ515294 | ﻿3.77e-64 | Bacteria; Firmicutes; Bacillales; Paenibacillaceae; Paenibacillus. | Choo et al. (2003) [54] |
| 4 | I | GA15030.A.B5 | 8/8 | *Polaromonas naphthalenivorans* | CP000529 | ﻿6.33e-106 | Bacteria; Proteobacteria; Betaproteobacteria; Burkholderiales; Comamonadaceae; Polaromonas | Copeland et al. (unpublished) |
| 5 | I | GA22030.A.G10 | 47/37 | *Azospirillum lipoferum* | AY786992 | ﻿2.07e-118 | Bacteria; Proteobacteria; Alphaproteobacteria; Rhodospirillales; Rhodospirillaceae; Azospirillum | Blaha et al. (2005) [55] |
| 6 | I | GA22075.A.C12 | 20/17 | *Azospirillum brasilense* | GQ161238 | ﻿5.19e-126 | Bacteria; Proteobacteria; Alphaproteobacteria; Rhodospirillales; Rhodospirillaceae; Azospirillum | Shukla (unpublished) |
| 7 | I | GA22030.A.C9 | 17/16 | *Rhodopseudomonas lichen* | AB241413 | ﻿1.59e-151 | Bacteria; Proteobacteria; Alphaproteobacteria; Rhizobiales; Bradyrhizobiaceae; Rhodopseudomonas | Xie et al. (unpublished) |
| 8 | I | GA04070.B.C9 | 3/1 | *Methylosinus trichosporium* | AF378724 | ﻿2.69e-123 | Bacteria; Proteobacteria; Alphaproteobacteria; Rhizobiales; Methylocystaceae; Methylosinus. | Auman et al. (2001) [56] |
| 9 | I | GA22100.A.H6 | 22/16 | *Xanthobacter autotrophicus* Py2 | CP000781 | 5.02e-119 | Bacteria; Proteobacteria; Alphaproteobacteria; Rhizobiales;Xanthobacteraceae; Xanthobacter | Gilbert et al. (unpublished) |
| 10 | I | GA16030.A.G1 | 39/39 | *Burkholderia vietnamiensis* | AM110707 | ﻿3.27e-160 | Bacteria; Proteobacteria; Betaproteobacteria; Burkholderiales; Burkholderiaceae; Burkholderia; Burkholderia cepacia complex. | Izumi (unpublished) |
| 11 | I | GA04070.B.A3 | 265/124 | *Ideonella dechloratans* | EU542578 | ﻿7.70e-143 | Bacteria; Proteobacteria; Betaproteobacteria; Burkholderiales; Ideonella. | Noar & Buckley (2009) [57] |
| 12 | I | GA17030.A.D3 | 73/58 | *Bradyrhizobium* sp. ORS391 | FJ347449 | ﻿1.00e-147 | Bacteria; Proteobacteria; Alphaproteobacteria; Rhizobiales; Bradyrhizobiaceae; Bradyrhizobium. | Nzoue et al. (2009) [58] |
| 13 | I | GA09100.A.A11 | 83/75 | *Vibrio diazotrophicus* | AF111110 | ﻿1.32e-95 | Bacteria; Proteobacteria; Gammaproteobacteria; Vibrionales; Vibrionaceae; Vibrio. | Wommack et al. (unpublished) |
| 14 | I | GA17030.A.G10 | 85/41 | *Teredinibacter turnerae* | CP001614 | ﻿2.55e-98 | Bacteria; Proteobacteria; Gammaproteobacteria; Alteromonadales; | Yang et al. 2009 [52] |
| Total |  |  | 688/450 |  |  |  |  |  |
