# Supplementary material for: Nitrogen Fixation in Denitrified Marine Waters
Source: PLoS One. 2011 Jun 7;6(6):e20539. doi: 10.1371/journal.pone.0020539 (PMC3110191; doi:10.1371/journal.pone.0020539)
Supplement: Table S4 — Distribution of the different OTUs found at each station and depth during the Galathea-3 cruise. (DOC) [file pone.0020539.s006.doc]

Table S4: Distribution of the different OTUs found at each station and depth during the Galathea-3 cruise.

| Station | Depth (m) | OUT 1 | OUT 2 | OUT 3 | OUT 4 | OUT 5 | OUT 6 | OUT 7 | OUT 8 | OUT 9 | OUT 10 | OUT 11 | OUT 12 | OUT 13 | OUT 14 |
| --- | --- | --- | --- | --- | --- | --- | --- | --- | --- | --- | --- | --- | --- | --- | --- |
| 2 | 60 |  | x |  |  |  |  |  |  | x |  |  | x | x |  |
| 4 | 50 |  | x |  |  |  |  |  |  |  | x | x |  |  |  |
| 4 | 70 |  | x |  |  |  |  |  | x | x | x | x | x | x | x |
| 8 | 30 | x |  |  |  |  |  |  |  |  |  |  |  |  |  |
| 9 | 100 |  |  |  |  |  | x |  |  |  | x | x | x | x | x |
| 11 | 80 |  |  |  |  |  |  |  |  |  | x | x | x |  |  |
| 15 | 20 |  |  |  |  | x | x |  |  |  |  |  |  | x | x |
| 15 | 30 |  |  |  | x |  |  |  |  |  | x | x |  | x | x |
| 16 | 30 |  |  |  |  | x |  |  |  |  | x |  | x |  |  |
| 16 | 100 |  |  |  |  |  |  | x |  |  |  |  |  | x | x |
| 17 | 30 |  |  |  |  |  |  | x |  | x |  |  | x | x | x |
| 17 | 200 |  |  |  |  | x |  |  |  | x | x | x |  | x | x |
| 22 | 30 |  |  | x |  | x |  | x |  |  |  |  | x |  |  |
| 22 | 50 |  |  |  |  |  | x |  |  |  |  |  |  | x | x |
| 22 | 75 |  |  |  |  |  | x |  |  |  |  |  |  | x | x |
| 22 | 100 |  |  |  |  |  |  |  |  | x |  |  | x |  |  |
