# Supplementary material for: Nitrogen Fixation in Denitrified Marine Waters
Source: PLoS One. 2011 Jun 7;6(6):e20539. doi: 10.1371/journal.pone.0020539 (PMC3110191; doi:10.1371/journal.pone.0020539)
Supplement: Table S5 — Comparison between water column N2 fixation rates obtained from the literature ([8], [10], [35], [59]–[64]) and values obtained in this study. The table shows previously published rates for unicellular diazotrophs as well as rates obtained during this study. Colonial diazotrophic cyanobacteria were not included in the table because they were not detected in the study area. The listed techniques are: ARA (Acetylene Reduction Assay), NA (Nitrogenase Activity) and 15N2 (Stable isotope tracer technique). Rates reported as hourly estimates (*) were transformed into daily rates regardless of potential daily periodicity in unicellular diazotrophs. (DOC) [file pone.0020539.s007.doc]

Table S5: Comparison between water column N_2_ fixation rates obtained from the literature [8;10;35;59-64] and values obtained in this study[[1]](#footnote-2).

| Technique | ρN_2_ (nmol N l^-1^ d^-1^) | ΣρN_2_ (μmol m^-2^ d^-1^) | Date of Sampling | Study Site and Reference |
| --- | --- | --- | --- | --- |
| ^15^N_2_ | 0.01 – 0.3 | 7.5 | 2005 | Peruvian upwelling (This study, KN182-9 cruise) |
| ^15^N_2_ | 0 – 14 | 679 | 2007 | Peruvian upwelling (This study, Galathea-3 cruise) |
| ^15^N_2_ | 0.15 – 0.31 |  | 2005 | North Pacific [59] |
| NA**[[2]](#footnote-3)**, ARA**[[3]](#footnote-4)** | 0.012 -0.072 | 2.2 | 2002 | North Pacific ALOHA [10] |
| ARA; ^15^N_2_ |  | < 50 | 2001-2002 | Western Tropical Atlantic [60] |
| ^15^N_2_ | 0.24 – 3.6 | 11 – 103 | 2001-2002 | North Pacific ALOHA [8] |
| NA; ARA ^15^N_2_ | 0.48 – 3.6 |  | 2001- 2002 | Tropical North Atlantic [10] |
| ^15^N_2_ | 0.00 – 0.55 |  | 2000-2001 | North Pacific ALOHA [61] |
| ^15^N_2_ | 0.38**[[4]](#footnote-5)** |  | 2001 | North Pacific ALOHA [62] |
| ^15^N_2_ | 0.34 – 2.28^d^ |  | 2000 | North Pacific ALOHA [63] |
| ^15^N_2_ |  | 30-910 | 2004 | South Pacific [64] |
| ^15^N_2_ | 0.72 – 1.08 |  | 2006 | Alpine Meromictic Lake (46°33′N, 8°43′E) [35] |

1. Colonial diazotrophic cyanobacteria were not included in the table because they were not detected in the study area. [↑](#footnote-ref-2)
2. Nitrogenase activity [↑](#footnote-ref-3)
3. Acetylene Reduction Assay [↑](#footnote-ref-4)
4. Rates reported as hourly estimates were transformed into daily rates regardless of potential daily periodicity in unicellular diazotrophs. [↑](#footnote-ref-5)
